# High-Amylose Sodium Carboxymethyl Starch Matrices: Development and Characterization of Tramadol Hydrochloride Sustained-Release Tablets for Oral Administration

**DOI:** 10.1155/2014/391523

**Published:** 2014-04-08

**Authors:** Teresa Nabais, Grégoire Leclair

**Affiliations:** Faculté de Pharmacie, Université de Montréal, P.O. Box 6128, Downtown Station, Montreal, QC, Canada H3C 3J7

## Abstract

Substituted amylose (SA) polymers were produced from high-amylose corn starch by etherification of its hydroxyl groups with chloroacetate. Amorphous high-amylose sodium carboxymethyl starch (HASCA), the resulting SA polymer, was spray-dried to obtain an excipient (SD HASCA) with optimal binding and sustained-release (SR) properties. Tablets containing different percentages of SD HASCA and tramadol hydrochloride were produced by direct compression and evaluated for dissolution. Once-daily and twice-daily SD HASCA tablets containing two common dosages of tramadol hydrochloride (100 mg and 200 mg), a freely water-soluble drug, were successfully developed. These SR formulations presented high crushing forces, which facilitate further tablet processing and handling. When exposed to both a pH gradient simulating the pH variations through the gastrointestinal tract and a 40% ethanol medium, a very rigid gel formed progressively at the surface of the tablets providing controlled drug-release properties. These properties indicated that SD HASCA was a promising and robust excipient for oral, sustained drug-release, which may possibly minimize the likelihood of dose dumping and consequent adverse effects, even in the case of coadministration with alcohol.

## 1. Introduction

Starch is a naturally occurring and a biodegradable polymer that is metabolized by the human body. Besides being nontoxic, starch is an abundant, cost-effective, and renewable material [1]. Due to these advantages, starches and modified starches have been widely and safely used in the food industry as thickeners, enhancers, of organoleptic properties, or texture modifiers and in the pharmaceutical industry as fillers, binders, disintegrants [2], and, more recently, as hydrophilic excipients for controlled drug-release. Numerous starch-modification methods, such as chemical [3], physical (i.e., gelatinization) [4], enzymatic [5], or a combination thereof, have been employed to produce new starch products with specific properties. Starch is a good candidate for chemical reaction or transformation because it is composed of amylose and amylopectin, two glucose polymers presenting three hydroxyl groups accessible as chemically active functional entities. Some of the modifications commonly employed to prepare starch derivatives are carboxymethylation, ethoxylation, and oxidation [6].

Substituted amylose (SA) has been introduced as a promising pharmaceutical excipient for sustained drug-release. SA matrix tablets have been prepared by direct compression, which is the easiest way to manufacture an oral dosage form and consists of dry mixing of drug and SA polymers followed by compression [7, 8]. The first SA polymer showing good properties as an excipient for sustained-release (SR) was produced by an etherification process using high-amylose corn starch and glycidol as the substituent and was referred to as SA,G-2.7, where G defines glycidol and 2.7 represents the degree of substitution (DS), expressed as the ratio of mole of substituent per kilogram of amylose [7, 8]. These properties included almost constant* in vitro* drug-release [7–9]; no significant influence of compression forces (CF) ranging from 0.5 to 5.0 tons/cm^2^ on the drug-release properties [9, 10]; large ranges of use for drug loading, drug solubility, and tablet weight (TW) [7, 11]; and unusually high crushing force values [10].

The use of sodium chloroacetate or chloroacetic acid in place of nonionic substituents like glycidol has been subsequently proposed to produce an excipient more readily acceptable by regulatory agencies for its nontoxicity [8, 12]. Sodium carboxymethyl starch with low-amylose content was already a well-known disintegrant agent in immediate-release (IR) tablets [13]. High-amylose sodium carboxymethyl starch (HASCA, where “HAS” means high-amylose substituted starch and “CA” defines the substituent used, herein chloroacetate), contrariwise, has been introduced as a promising pharmaceutical excipient for oral SR tablets prepared by direct compression [12]. Contramid, a cross-linked high amylose starch, has already been used in tablets as a retarding agent [14].

After being initially produced on laboratory scale, the polymer was obtained on an industrial pilot scale. However, these latter batches of polymer were unsuitable for tableting and sustained drug-release due to inadequate physical properties. For this reason, the material was spray-dried (SD) to obtain a compressible excipient. This procedure, which has been described elsewhere [6], resulted in SD HASCA, the polymer used in the current study.

Studies on the* in vitro* release characteristics of acetaminophen from tablets composed of SD HASCA, drug, and sodium chloride have demonstrated the suitability of this polymer as a sustained drug-release excipient. No burst effect was observed, even in the presence of large amounts of soluble sodium chloride in the formulation [15, 16]. Moreover, the release of acetaminophen from optimized SD HASCA formulations is not significantly influenced by the variations of pH and residence time in acidic media [16]. These prior results suggest that drug-release from SD HASCA tablets may not be affected by intra- and intersubject variations in gastric pH or gastric residence time. Acetaminophen (phenol, pKa 9.51) and tramadol hydrochloride (tertiary amine, pKa 9.41) are completely nonionized and completely ionized at physiological pH, respectively. Moreover, a first* in vivo* study where the same optimized SD HASCA formulation was administered orally to healthy human subjects has demonstrated extended acetaminophen plasma levels and demonstrated that the gel formed by the polymer was strong enough to resist both the powerful peristaltic contractions normally occurring in the fasted state and biodegradation in the intestine [16]. Other features of SD HASCA that confirm the potential of this polymer for sustained drug-release include its excellent binding properties in the absence of binders, which resulted in very high crushing forces [6, 15]. A strong dependence of drug-release on TW has also been found, corresponding to a TW rise to an increased total release time [15].

Tramadol is a synthetic, centrally acting analgesic agent with a dual mechanism of action [17]. It has proven to be effective in the treatment of moderate to moderately severe pain without causing severe side effects and organ damages [18]. For the treatment of chronic pain, frequent oral administration of IR tramadol is required (at least four times daily) because of its mean elimination half-life, which can vary from 5 to 7 h in humans [19]. In adults and adolescents, the usual oral dosage regimen is 50 to 100 mg every 4 to 6 hours, with a maximum dosage of 400 mg/day [17]. Consequently, oral formulations of tramadol that provide a more gradual release of the drug have been developed with the aim of reducing the frequency of administration, minimizing acute adverse effects, and thus improving the patient compliance [20, 21]. After oral administration, tramadol is rapidly and almost completely absorbed. Its bioavailability of 70% after a single administration can be attributed entirely to extensive first pass metabolism [22]. However, at steady state, the bioavailability of a SR formulation has been shown to be 100% relative to an IR formulation [23], which represents another advantage of SR formulations of tramadol. Bioequivalence after dose adjustment between IR and SR formulations has been demonstrated [19]. Tramadol hydrochloride is readily soluble in water and ethanol and hence judicious selection of release retarding excipients is necessary to achieve an extended* in vivo* input rate of the drug.

SR formulations, especially those designed for once-daily administration, contain amounts of drug that can be potentially risky if released as a burst, commonly called dose dumping. Tramadol hydrochloride may cause severe side effects if high plasma concentrations are reached quickly. Inadvertent or intentional misuse of a SR formulation, with consequent dose dumping, may include breaking, crushing tablets and simultaneous coingestion of the medication with high doses of ethanol. Therefore, the robustness of SD HASCA tablets in an ethanolic medium and their crushing strength values are important factors to take into account.

In this study, the* in vitro* release characteristics from matrix tablets comprising tramadol hydrochloride as the model drug and SD HASCA as the only SR excipient were evaluated in a series of buffers simulating the pH of the fluids throughout the gastrointestinal tract. Tablets intended to twice-daily and once-daily administration were developed. These tablets contained 100 mg and 200 mg of drug, respectively. The dissolution profiles of these tablets were compared with those of marketed tramadol hydrochloride SR formulations of the same drug content. The resistance of the gel formed by SD HASCA to an alcoholic medium was also investigated.

## 2. Materials and Methods

### 2.1. Materials

Amorphous HASCA (Eurylon 6, batch 3910, ref. 799511) was provided by Roquette Frères (Lestrem, France) and contained approximately 60% of amylose and 40% of amylopectin. The DS was equal to 0.045 (number of moles of substituent/number of moles of anhydroglucose) [15]. Tramadol hydrochloride was purchased from Jubilant Organosys Ltd. (Nanjangud, Mysore, India). Commercially available tramadol tablets Tridural 100 mg and 200 mg, Ralivia 200 mg, Zytram XL 200 mg, Topalgic LP 100 mg and 200 mg, and Contramal LP 200 mg were purchased from pharmacies in Canada and in France. All chemicals were of reagent grade and were used without further purification.

### 2.2. Preparation of HASCA Tablets

Amorphous HASCA, as provided by its manufacturer, did not possess the required binding and SR properties to be used as a suitable material for SR tablets prepared by direct compression. Therefore, this polymer was spray-dried as described by Brouillet et al. [6] by first preparing a suspension of HASCA in a mixture of water and ethanol (final water/ethanol ratio of 1.33, 4 mL of ethanol/g of HASCA) and then spray-drying using a Büchi B-290 Mini-Spray-Dryer (Flawill, Switzerland).

Tablets were prepared by direct compression using a 30-ton manual hydraulic press (referred to herein as HP, C-30 Research & Industrial Instruments Company, London, U.K.) equipped with round flat-faced tooling (diameter 12.6 mm) or a single-stroke press machine (referred herein as SSP, Manesty F3 Machine, Manesty Machines Ltd., Liverpool, UK) also equipped with round flat-faced tooling (diameter 11.1 mm). In the HP, compression force was adjusted to target a specific CF, and in the SSP, compression force was adjusted to target specific thickness. SD HASCA tablets of different total weights containing different percentages of polymer and drug were produced as described in Table 1.

### 2.3. Tablets Characterization and* In Vitro* Drug-Release Evaluation

The crushing forces (Newton) of SD HASCA tablets were measured using a hardness tester (Pharma Test Type PTB 301, Hainburg, Germany) and their thickness using an electronic caliper (Starrett, 799A Series).

The* in vitro* drug-release properties of SD HASCA SR tablets in simulated gastric fluid (stage I) and simulated intestinal fluids (stage II and stage III) were assessed using a USP apparatus 2 (900 mL, 37°C, 50 rpm) [24].

During stage I, the tablets were exposed to hydrochloric acid buffer (pH 1.2), simulating gastric pH (SGF) for 1 h. During stage II, the tablets were exposed to phosphate buffer (pH 6.8) simulating jejunum pH (SIF I) for an additional 3 h. Finally, the tablets were exposed to a second phosphate buffer (pH 7.4) simulating ileum pH (SIF II) until the end of the assay (total time of 24 h). All standard buffer solutions were prepared according to the USP.

In addition, dissolutions tests in a 40% ethanol in water solution were performed to assess the release properties of a SD HASCA formulation and a commercial formulation with the same drug content and the same frequency of administration. This percentage of ethanol was chosen because it represents a standard strength of alcoholic spirits [25]. Tests in alcohol were carried out during 7 h.

**Figure 1:**
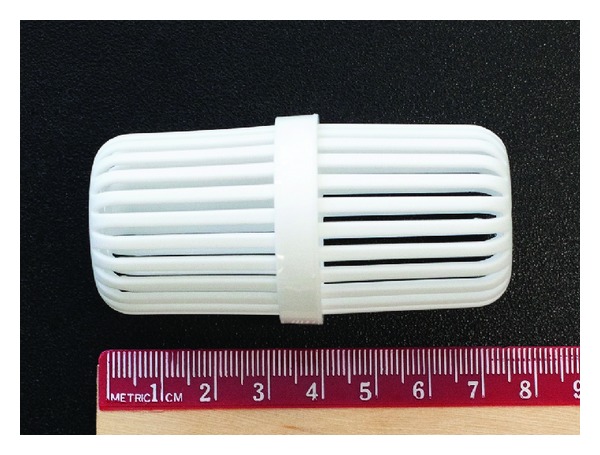
Plastic cage used in the dissolution experiments to avoid eventual adhesion to the vessel or floating.

To avoid eventual floating to the surface of the medium or adhesion to the vessel, each tablet was placed inside a perforated cylindrical plastic cage (Figure 1). The cage had enough internal space for drug-release to occur from all sides of the matrix and its perforations were big enough not to interfere with the circulation of the medium inside the dissolution vessel but, on the other hand, were small enough to keep the tablets inside.

According to USP specifications, the paddle should be positioned at a distance of 25 mm of the inside bottom of the vessel. Due to the presence of the cage at the bottom of the dissolution vessel, the paddle was positioned at a distance of 75 mm of the inside bottom of the vessel. After each sampling, an equivalent volume of fresh medium (10 mL) was added. Each experiment was performed in triplicate. The occurrence of macroscopic transformations in the matrix tablets and their eventual influence on the release profiles were registered. Samples were filtered through syringe filters (0.45 mm, nylon). Tramadol hydrochloride release was followed by UV spectrometry (271 nm). A calibration curve was prepared for each of the three mediums (pH 1.2, 6.8, and 7.4) as well as for the ethanolic medium and anew before the measurement of the samples resulting from each dissolution test. The drug-release results are expressed as cumulative percentage (%) as a function of time (h).

The* in vitro* release properties of twice-daily and once-daily commercial formulations with 100 mg or 200 mg of tramadol hydrochloride were also evaluated using the same experimental conditions, so as to provide a direction regarding the desired release times. The twice-daily dosage forms tested were Topalgic LP 100 mg, Topalgic LP 200 mg, and Contramal LP 200 mg, and the once-daily dosage forms tested were Tridural 100 mg, Tridural 200 mg, Ralivia 200 mg, and Zytram XL 200 mg.

### 2.4. Modifications to the United States Pharmacopoeia (USP) Method

In the first* in vitro* studies on the release of tramadol hydrochloride from SD HASCA matrix tablets some formulations showed a propensity to float or to adhere to the bottom of the vessel. This propensity was higher as the concentration of tramadol hydrochloride increased. This situation makes the tablets very susceptible to large variations in hydrodynamic conditions that prevail in a dissolution vessel. In addition, the tablets floating at the surface of the dissolution medium or adhering to the glass surface had a lower surface area in contact with the medium. This situation can affect drug-release from the tablets, as the exposed surface area is a major factor determining release kinetics both in erosion-controlled as well as diffusion-controlled systems [26].

The use of perforated cylindrical plastic cages placed below the paddles in the dissolution vessels appeared to be successful in preventing adhesion to the vessel and floating. This solution was chosen in detriment of the use of USP apparatus I (basket) as the use of this apparatus can interfere with the free swelling of the matrix. However, the paddle relative position had to be at a higher height compared to what is settled by the USP because of the size of the cage. Hence, the influence of the paddle position on drug-release was evaluated using acetaminophen 700-mg tablets. These tablets were composed of acetaminophen (40%) and HPMC (60%). Equivalent dissolution (*f*_2_ = 99.77) was achieved when the paddle was positioned according to USP specifications (25 mm) and when the paddle was positioned higher (75 mm).

### 2.5. Statistical Analysis and Formulations Comparison

The arithmetic average and the standard deviation of the cumulative % of drug released at predetermined time intervals were calculated.

Dissolution profiles were compared using the similarity factor (*f*_2_), according to the following equation proposed by Moore and Flanner [27]:

(1) $$\begin{array}{c} f_{2}=50\times \log\left\{{\left[1+\left(\frac{1}{n}\right)\overset{n}{\underset{t-1}{\sum }}{\left(R_{t}-T_{t}\right)}^{2}\right]}^{-0.5}\times 100\right\}, \end{array}$$

where *R*_*j*_ and *T*_*j*_ are the percent dissolved of the reference and test products at each time point *j* and *n* is the number of pool points. Two dissolution profiles are similar if *f*_2_ is between 50 and 100 according to the Food and Drug Administration (FDA) and the European Agency for the Evaluation of Medical Products (EMEA). However, the closer to 100 (%)*f*_2_, the more similar the dissolution profiles are. The dissolution profiles were also compared using the values of the time to release 50% (T50%) and to release 90% of drug (T90%).

## 3. Results and Discussion

### 3.1. Measurement of Tablet Crushing Strengths in Order to Guarantee the Reproducibility of the Spray-Drying Method

The reproducibility of the method used to transform amorphous HASCA into SD HASCA was assessed through the evaluation of tablet crushing forces for different batches. SD HASCA tablets weighting 200 mg were prepared by direct compression. The excipient was compressed at a CF of 2.5 tons/cm^2^ using a HP using a dwell time of 20 seconds. The values of the crushing forces of 200-mg tablets produced from different batches of SD HASCA varied between 124.9 ± 10.5 and 148.5 ± 3.2 Newton (arithmetic averages ± standard deviations). Therefore, the crushing forces were considered reproducible. As non-SD HASCA particles did not possess any biding properties and the SD procedure resulted in a decrease in particle size of the polymer, it has been hypothesized that these unusual high crushing strength values are due, in part, to the particle size reduction, which corresponds to a higher surface area of the particulate product and provides a higher number of binding points. In addition, it is believed that the combination of water and ethanol in the SD process has a plasticizer effect, causing partial melting of the polymer and particle rearrangement under compression [6].

### 3.2. Formulation Screening and Development of Twice-Daily and Once-Daily SD HASCA Formulations with 100 mg and 200 mg of Tramadol Hydrochloride

The values of the T50% and T90% of tramadol hydrochloride from tablets of different total weights and containing different percentages of polymer and tramadol hydrochloride (compressed with the HP) are summarized in Table 2. Increasing the percentage of tramadol hydrochloride in the tablet led to an increase in the drug-release rate for all TWs, which is a common observation for hydrophilic matrices. In addition, the total release time increased significantly as a function of TW. The T50% was, though, less affected by TW than the T90%.

When immersed in the dissolution mediums, a very rigid gel layer with high mechanical strength in the swollen state formed on the surface of the tablets. This strong gel layer, which is formed by hydrogen bonding between the hydroxyl groups of the soluble gelled starch chains, has been documented before for SD HASCA [15]. In addition, its formation has been related to the pH-independent SR behaviour of high-amylose sodium carboxymethyl starch produced at low DS, as it is the case of SD HASCA [28]. The gel layer formed by SD HASCA showed a lower degree of swelling when compared to the gel layer formed by hydroxypropylmethylcellulose (HPMC) (results not shown). The most important aspect of the release mechanism from tablets constituted by hydrophilic polymers is known to be the formation of a gel layer around the dry core of the matrix in response to water penetration. This gel layer hinders fast inward medium penetration and outward drug diffusion. Phenomena that govern gel layer formation and, therefore, drug-release, are penetration of the dissolution medium into the matrix, resulting in polymer hydration and swelling (relaxation process), drug dissolution and diffusion through the layer of swollen polymer, and erosion on the surface of the matrix. The formation of the gel layer is caused by the polymer-state transition. The gel layer acts like a barrier against the fast release of drugs whilst controlling at the same time the penetration of aqueous medium and the diffusion of drug. As the size of the internal dry and/or partially hydrated core of the tablets increases as a function of TW for the same concentration of drug, if the core is considered a drug reservoir, the larger is the internal reservoir the longer is the time to empty it, which explains the increase of the total release time as a function of TW. Besides, as demonstrated in a previous study, augmenting TW heightens the contribution of diffusion to the detriment of erosion as the main mechanism controlling drug-release, leading to a decline in the drug-release rate [15]. Furthermore, a combination of two release mechanisms has been described as controlling the kinetics of drug-release from SD HASCA matrices: drug diffusion through the gel layer after drug dissolution (Fickian diffusion) and polymer disentanglement and erosion (case II relaxational release) [15]. Fickian diffusion release occurs by diffusion of drug molecules due to a concentration gradient. Case II relaxational release is the mechanism associated with polymer-state transition from the glassy, a solid state (dry polymer), to the rubbery state, an expanded and flexible state, which is linked with the polymer swelling process [29]. Similar conclusions were reported for the transport of acetaminophen from nonionic SA matrices [9, 11]. It is known that drug concentration and thickness of the gel layer are the factors governing drug-release [30]. At lower drug concentrations, that is, higher polymer concentrations, diffusion is the main transport mechanism, and thus the total release time was longer. However, as drug concentration increases the drug-release rates also increase. Tramadol hydrochloride is freely soluble in water and thus dissolves quickly in aqueous medium. Its high solubility along with an increase in drug loading resulted in higher drug concentration in the gel. In addition, the high hydrophilicity of tramadol hydrochloride promotes the penetration of the dissolution medium into the matrices. Above a certain drug-loading threshold, the quantity of absorbed water may be such that the water-polymer interactions become superior to the polymer-polymer interactions, causing chain disentanglement and polymer dissolution [31]. As a result, progressive erosion appears on the surface of the tablets, leading to a decrease in the thickness of the gel layer and an increase in the drug-release rate. Indeed, it has been reported that the gel strength is an essential factor in the matrix performance and is controlled not only by the viscosity and chemical structure of the rubbery polymer but also by its concentration [30]. Therefore, lower SD HASCA concentrations in the tablet led to lower gel strengths and thus to higher release rates, owing to higher erosion. Besides, it can be hypothesized that at certain drug/polymer ratio the release process will become governed mainly by erosion. However, opposite results have been observed for tablets containing SD HASCA, acetaminophen, and sodium chloride. In this case, an increase in drug loading corresponded to an increase in total release time, until a certain level where erosion occurred [15]. It is important to take into consideration the higher solubility of tramadol hydrochloride compared to acetaminophen. Indeed, when formulating SD HASCA matrices, not only the concentration of the components has to be taken into account but also their solubility, nature, and interactions between them.

As the values of the time to release 25% of drug (T25%) for different TWs were similar, especially for the 400-mg tablets, these values are not presented. The T25% corresponds usually to the burst effect and depends on the amount of drug on the table surface available for immediate dissolution and release in the medium.

**Figure 2:**
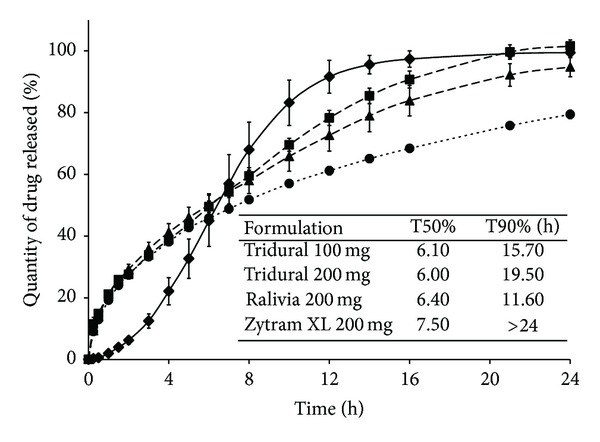
Cumulative % of tramadol hydrochloride released* in vitro* from once-daily commercial formulations (■: Tridural 100 mg, ▲: Tridural 200 mg, ◆: Ralivia 200 mg, and ●: Zytram XL 200 mg).

The formulation screening allowed the investigation of the most adequate total weights for four formulations with SD HASCA as the SR excipient: two twice-daily with 100 mg and 200 mg of tramadol hydrochloride and two once-daily with 100 mg and 200 mg of tramadol hydrochloride. In order to select these formulations, the* in vitro* drug-release profiles from tablets with 100 mg and 200 mg of drug, which were produced using the HP to study the influence of drug content and TW on the dissolution rate, for the formulation screening described above, were compared to the* in vitro* drug-release profiles of commercially available twice-daily and once-daily tramadol hydrochloride formulations with the same drug content. The release profile from the tablets with a total weight of 700 mg, containing 100 mg of drug and compressed with the HP at 2.5 tons/cm^2^ for 20 seconds, which were prepared to study the influence of the CF on the release rate, was also compared to the release profile of commercial formulations.  Figure 2 shows the drug-release profiles from the commercial once-daily dosage forms.

The values of T50% and T90% for the tablets weighting 500 mg with 200 mg of tramadol hydrochloride were 1.9 and 6.7 hours (Table 5), very similar to the values for Topalgic LP 200 mg, 2 and 6.9 hours, respectively (Table 3). In addition, the *f*_2_ resultant from the comparison of their profiles resulted in value of 86.54. Consequently, the 500-mg tablet with 200 of drug was chosen as the twice-daily formulation with 200 mg of drug.

An evaluation of the release characteristics of a formulation corresponding to a total weight of 450 mg with 100 of drug was also performed to determine if its release characteristics approached better the ones of the 500-mg tablet with 200 mg. The T50% and T90% for this formulation were 1.9 and 6.4 hours (Table 5), very similar to values of the selected twice-daily SD HASCA formulation with 200 mg, and more similar than the T50% and T90% for the 400-mg and 500-mg tablets with 100 mg of drug. In addition, the *f*_2_ results also showed that the 450-mg tablet with 100 mg of drug presented release characteristics more similar to the selected twice-daily formulation with 200 mg of tramadol hydrochloride (*f*_2_ equal to 89.23) and to Topalgic LP 100 mg (*f*_2_ equal to 72.71), which led to the selection of the formulation weighting 450 mg as the twice-daily SD HASCA formulation with 100 mg. Close similarity between both the twice-daily and the once-daily developed formulations with different contents of drug was desired.

Given that SD HASCA formulations were produced for oral administration, the size of the tablets had to be taken into consideration when producing once-daily formulations. Consequently, an increase in tablet size was not considered and the tablet weighting 800 mg with 200 mg of drug was chosen as the once-daily formulation with 200 mg of tramadol hydrochloride, even considering that the release rate from this tablet was faster than the rate from Tridural 200 mg (*f*_2_ equal to 55.83). In addition, even though the release from the tablets weighting 800 mg with 100 mg of tramadol hydrochloride was slower and more similar to Tridural 100 mg (*f*_2_ equal to 68.1) than the tablet weighting 700 mg with the same drug content (*f*_2_ equal to 53.09), the latter had a profile more similar to the tablet with a total weight of 800 mg and 200 mg of tramadol hydrochloride (*f*_2_ equal to 82.39), the selected once-daily SD HASCA formulation with 200 mg of drug. Therefore, the 700-mg tablet with 100 mg of drug was chosen as the optimal once-daily formulation with 100 of tramadol hydrochloride.

A pH gradient was used instead of a unique buffer or distilled water because it provides a more accurate simulation of the environment that an oral dosage form encounters when transiting through the gastrointestinal tract. Besides, studies have shown that* in vivo*-*in vitro* correlations (IVIVC) are improved when the dissolution tests are carried out in a pH gradient rather than in distilled water [32].

Studies have demonstrated that the DS has a significant influence on the structural, physicochemical, and drug-release properties of high-amylose carboxymethyl starch [28, 33]. These studies have suggested that high-amylose sodium carboxymethyl starches produced at lower DS are preferably suitable for use as an excipient for SR formulations, showing extended-release in both acidic and alkaline dissolution media, while high-amylose sodium carboxymethyl starch at higher DS (referred to as CM-HAS) can be used as an excipient for delayed-release, since the drug-release rate in acidic medium is significantly lower than in alkaline medium [28, 33]. As a result, the potential of this polymer produced at higher DS as an excipient for formulations with gastroresistant properties of a variety of drugs, including bioactive agents, has been investigated [34, 35]. The polymer used in this study was produced at a lower DS and, therefore, shows SR properties in both acid and alkaline environment.

### 3.3. Decrease of the Tablet Surface Area in Order to Increase the* In Vitro* Release Time of Tramadol Hydrochloride from Once-Daily and Twice-Daily SD HASCA Matrix Tablets

The comparison between the T50% and T90% from the once-daily SD HASCA formulations with 100 mg (4 and 14.4 h) and 200 mg of tramadol hydrochloride (4.1 and 16.4 h), prepared using a HP (Table 5), and the T50% and T90% from Tridural 100 mg (6.1 and 15.7 h) and Tridural 200 mg (6 and 19.5 h), the once-daily commercial dosage forms (Table 3), show that the release rates from the once-daily SD HASCA tablets were slightly higher than the release rates from the commercial tablets. Closer values were, however, found for the tablets intended for twice-daily administration.

The ultimate purpose of the developed SD HASCA SR formulations is oral administration in humans. Tablet size, shape, and geometry are important factors to take into consideration when designing medication to be administered by this route, since they determine the level of patient comfort during oral administration, and thus compliance. Besides, it has been shown that tablet size, shape, and surface area may affect drug-release profiles [36, 37] and can, therefore, be used for modulation of drug-release rate. A study on the influence of tablet surface area/volume (SA/V) ratio on drug-release from controlled-release matrix tablets containing HPMC indicated a direct relationship between SA/V ratios and drug-release rate. Utility of SA/V to influence drug-release was demonstrated by altering tablet shape to adjust SA/V [36]. The importance of this finding is related to the possibility of achieving optimal drug-release profiles without further modification of a formulation, by simply choosing an appropriate SA/V ratio for a tablet.

Based on the higher comfort for the patient when swallowing tablets with narrower diameters and the direct link found between surface area and drug-release rate, the dimensions of the selected SR SD HASCA formulations were changed so as to decrease their diameter, decreasing the tablet SA/V ratio. To achieve these changes, the tablets were compressed using a SSP machine instead of the 30-ton manual HP. This compressing machine produced tablets with an average diameter of 11.1 mm instead of 12.6 mm and, consequently, tablet heights were thicker.

When using the SSP, the lower plunger nuts were adjusted so as to achieve a target average thickness of 7 mm for the once-daily tablet with 100 mg of drug(instead of 4.4 mm of the tablets produced with the HP), 7.4 mm for the once-daily with 200 mg of drug (instead of 4.8 mm of the tablets produced with the HP), and 4.6 mm for the twice-daily tablets with 100 mg and 5.2 mm for the twice-daily tablets with 200 mg (no thickness measurements had been made with the HP). As the number of sample tablets was small, it was not possible to present the values of the standard deviations of the tablet thickness.

The* in vitro* drug-release characteristics from the tablets produced with the SSP were then evaluated under the same pH gradient dissolution conditions. Even though the twice-daily SD HASCA formulations presented a release profile close to the profile of the commercial ones, the same changes in surface area were studied so as to develop all the four formulations under the same conditions and make them all more easily swallowable.

#### 3.3.1. Preliminary Crushing Strengths Measurements

The crushing strengths of 700-mg and 800-mg tablets with 100 mg and 200 mg of tramadol hydrochloride, respectively, compressed with the HP were measured. The designed procedure was to apply a CF starting at 1 tons/cm^2^ and to increase it gradually up to 2.5 tons/cm^2^. The tensile strength of these tablets was so high that none of them broke after being submitted to the hardness tester. The hardness tester allowed a maximum measurable crushing force of 300 N. Therefore, no further measurements were necessary. The very high crushing strengths found for these tablets support the theory, described above, of the occurrence of particle rearrangement and partial melting of the polymer under compression, resulting in the densification of the matrices. The high crushing strength values are an advantage when considering industrial manufacture because they ensure batch reproducibility throughout the process.

#### 3.3.2. Influence of CF on Tramadol Hydrochloride Release from SD HASCA Tablets

The influence of CFs ranging between 1 and 2.5 tons/cm^2^ on the drug-release properties from SD HASCA matrix tablets weighting 700-mg tablets with 100 mg of tramadol hydrochloride and compressed using a 30-tons manual HP was also investigated. This range of CFs was selected because it covers the normal range of CF employed at the industrial level. The T50% and T90% for the tested CFs and the values of the *f*_2_ between tablets compressed at different CFs are represented in Table 4. Between 1 and 2.5 tons/cm^2^, CF did not significantly influence drug-release from SD HASCA matrix tablets. Similarly, the drug-release was not significantly influenced by the compression force for tablets containing 32.5% of SD HASCA, 40% of acetaminophen and 27.5% of sodium chloride compressed at a total weight of 400 and 600 mg.

These consistent results show that the independence between drug-release from SD HASCA matrices and the CF used is not affected by the different composition of the polymer, the final ethanol/HASCA ratio, and the substitution of sodium chloride for tramadol hydrochloride as the electrolyte in the formulation. Ungur et al. [12] have already noted that, in the case of lab-scale HASCA, CF influenced microporosity but did not alter the drug-release rate. A study using formulations composed of SD HASCA, acetaminophen, and sodium chloride, where the relationship between TW and CF versus tablet thickness (TT) was investigated to understand the good biding properties of SD HASCA, suggested that an intense densification of the matrices occurred and was the same for CFs ranging from 1 to 2.5 tons/cm^2^. In this case, it was also demonstrated that CF does not considerably influence TT [15]. This peculiar mechanism of densification, that is, sintering by viscous flow and melting under compression, has been demonstrated by scanning electron microscopy (SEM) and porosimetry for matrices composed of SA,G-2.7 [10]. Such very low porosity might explain why CF does not influence the drug-release rate from tablets with SD HASCA or SA,G-2.7 as the SR excipient. As opposed to SA,G-2.7, no influence of CFs on the amplitude of the burst effect as well as on the time-lag has also been observed for SD HASCA tablets.

The fact that the CF applied did not significantly influence drug-release is important since it indicates that the unknown CF exerted by the SSP machine may not have a significant influence on the release of tramadol hydrochloride from SD HASCA tablets compressed with this machine. This finding and the very high crushing strengths further illustrate the utility of SD HASCA as a directly compressible excipient for oral sustained drug-release and assure the reliableness of the SSP.

#### 3.3.3. *In Vitro* Release of Tramadol Hydrochloride from Optimal Twice-Daily and Once-Daily SD HASCA Matrix Tablets

**Figure 3:**
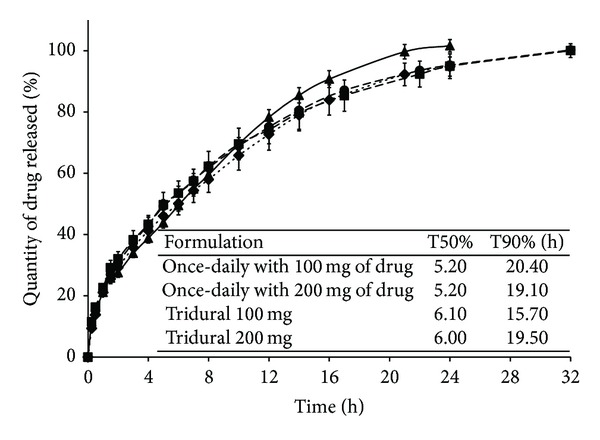
Cumulative percentage of tramadol hydrochloride released* in vitro* from once-daily SD HASCA formulations, compressed using a single-stroke press machine (SSP), and once-daily commercial formulations (■: 700-mg tablets with 100 mg of drug, ●: 800-mg tablets with 200 mg of drug, ▲: Tridural 100 mg, and ◆: Tridural 200 mg). Tests were performed with the tablets placed inside a cage. *f*_2_ values: 70.66 between 700-mg tablets with 100 mg and Tridural 100 mg, 79.80 between 800-mg tablets with 200 mg and Tridural 200 mg, 93.14 between 700-mg tablets with 100 mg, and 800-mg tablets with 200 mg.

As shown in Figure 3, the 700-mg SD HASCA tablets (once-daily) with 100 mg of drug compressed with the SSP machine had a slightly lower release than the once-daily commercial formulations after 10 hours of release. At this stage of experimentation, this does not represent a problem given that one does not know yet how these SD HASCA dosage forms will behave* in vivo*.

Decreasing the diffusion surface area using the SSP led to a decrease in the release rate and consequently to an improvement of the similarity of the 700-mg tablet with 100 mg of drug to Tridural 100 mg, when compared with the same formulation compressed with the HP (*f*_2_ equal to 70.66 for the SSP versus 53.09 for the HP). In the same way, the *f*_2_ for the 800-mg tablet with 200 mg of drug compressed with the SSP machine was relatively higher than the *f*_2_ for the 800-mg tablet with 200 mg of drug compressed with the HP, when both formulations were compared to Tridural 200 mg (*f*_2_ equal to 79.80 for the SSP versus 55.83 for the HP). The *f*_2_ between the once-daily SD HASCA with 100 mg and the once-daily SD HASCA with 200 mg formulations compressed with the SSP was 93.14, much superior to the value between the commercial formulations, that is, 69.67.

**Figure 4:**
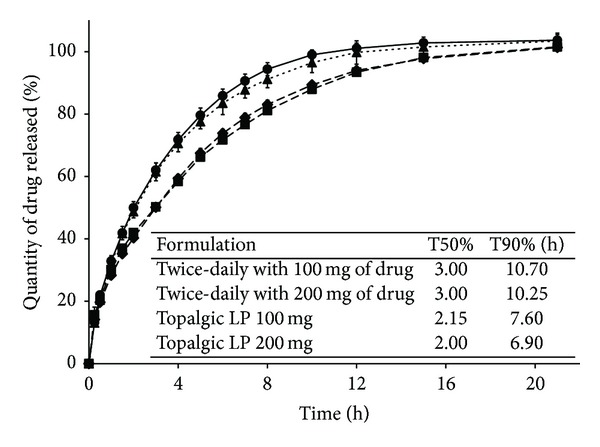
Cumulative percentage of tramadol hydrochloride released* in vitro* from twice-daily SD HASCA formulations, compressed with the single-stroke press machine, and twice-daily commercial formulations (◆: 450-mg tablets with 100 mg of drug, ■: 500-mg tablets with 200 mg of drug, ▲: Topalgic LP 100 mg, and ●: Topalgic LP 200 mg). Tests were performed with the tablets placed inside a cage. *f*_2_ values: 54.52 between 450-mg tablets with 100 mg and Topalgic LP 100 mg, 52.36 between 500-mg tablets with 200 mg of drug and Topalgic LP 200 mg, and 88.01 between 450-mg tablets with 100 mg and 500-mg tablets with 200 mg of drug.

Figure 4 shows that the release from both the 450-mg tablets with 100 mg of drug and the 500-mg tablets with 200 mg of drug, compressed with the SSP, was slower than the release from the commercial formulations with 100 mg and 200 mg (*f*_2_ equal to 54.52 for twice-daily SD HASCA 100 mg and Topalgic LP 100 mg and equal to 52.36 for twice-daily SD HASCA 200 mg and Topalgic LP 200 mg). The *f*_2_ regarding the similarity between the twice-daily SD HASCA formulations compressed with the SSP (88.01) and the *f*_2_ regarding the similarity between commercial formulations (85.06) were nearly identical. The differences between the release profiles from the two types of twice-daily formulations, commercial and SD HASCA, were not an issue, since the main goal of the present work was essential to prove the SR properties of the developed SD HASCA formulations and to develop once-daily and twice-daily SD HASCA formulations with similar release profiles for both dose strengths (100 mg and 200 mg) and type of formulation (once-daily and twice-daily). Besides, the commercial formulations were mainly a guide to remain within reliable release profiles for the 12-hour and the 24-hour release formulations. Therefore, a strict similarity was not required between SD HASCA and marketed formulations.

As it can be observed in Figures 3 and 4, the release profiles from SD HASCA tablets can be divided into two stages, which are typical of hydrophilic matrices. First, a burst effect occurs. This burst of drug corresponds to the rapid dissolution and release of drug from the tablet surface, whilst the viscous gel layer forms around the dry core of the matrix tablet. Thereafter, the release rate decreases gradually until the end of the dissolution process. This decrease in release rate corresponds to an increase of the diffusion pathway that drug molecules have to traverse, owing to the progressively forming gel barrier. This release behaviour is characteristic of a mainly diffusion-controlled mechanism [38].

Table 5 shows a clear decrease in the rate of drug-release, given by the T50% and T90%, from twice-daily and once-daily SD HASCA tablets compressed using the SSP when compared to the tablets compressed with the HP. In the same way as it was observed for the tablets compressed with the HP, the T50% was less affected by TW than the T90% when the tablets were compressed using the SSP. The* in vitro* release rates from the optimized twice-daily and once-daily SD HASCA formulations were in agreement with documented preferred profiles [39].

### 3.4. *In Vitro* Release of Tramadol Hydrochloride from SD HASCA Tablets under Ethanolic Conditions

**Figure 5:**
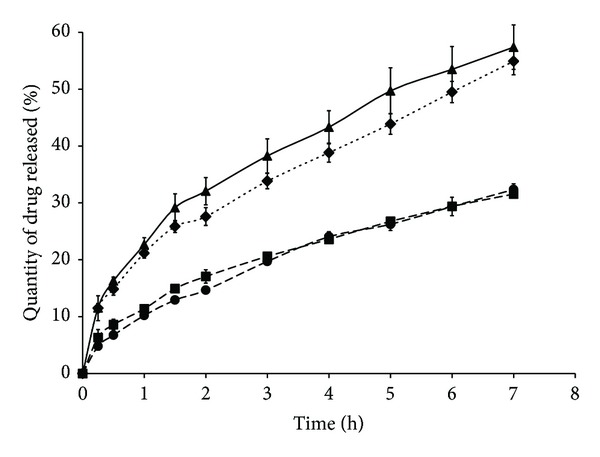
Cumulative percentage of tramadol hydrochloride released* in vitro* from once-daily SD HASCA tablets with 100 mg of drug and once-daily commercial formulations with 100 mg of drug in a pH gradient versus a 40% ethanol hydroalcoholic medium (▲: SD HASCA with 100 mg in a pH gradient, ◆: Tridural 100 mg in a pH gradient, ●: SD HASCA with 100 mg in a 40% ethanolic medium, and ■: Tridural 100 mg in a 40% ethanolic medium). *f*_2_ values: 70.66 between 700-mg tablets with 100 mg of drug and Tridural 100 mg in pH gradient, 88.51 between 700-mg tablets with 100 mg of drug and Tridural 100 mg in a 40% ethanol hydroalcoholic medium.

The results of the* in vitro* release of tramadol hydrochloride from once-daily SD HASCA tablets with 100 mg of tramadol hydrochloride and from Tridural 100 mg (once-daily) in a hydroalcoholic medium with 40% ethanol, performed to address safety issues related to simultaneous administration of SD HASCA tablets and ingestion of alcohol, are presented in Figure 5. The same figure shows the release profiles from the same formulations in a pH gradient. The rate of release of tramadol hydrochloride from SD HASCA tablets decreased under ethanolic conditions when compared to the rate of release in a pH gradient. SD HASCA formed a very hard gel in the hydroalcoholic medium, which retained SR properties independently of the alcoholic environment. The same decline in the rate of release was observed for Tridural 100 mg. The SD HASCA and the commercial formulations demonstrated very similar profiles (*f*_2_ = 88.51).

It has been reported that* in vitro* dissolution experiments in 40% ethanol hydroalcoholic mediums over a 2-hour period are expected to be representative of the most extreme alcoholic conditions likely to be encountered* in vivo*, because of to the dilution of the ethanol in the gastrointestinal fluids [25]. Yet, the dissolution tests in alcohol were carried out during a 7-hour period to better distinguish the different drug-release profiles between formulations and between dissolution mediums.

The decline in the release rate may be explained by a possible susceptibility of the solubility of SD HASCA, the SR agent in our tablets, and Contramid (a cross-linked starch), the SR agent in Tridural, to high-alcohol concentrations. Indeed, it has been shown that, in heated aqueous solutions containing some alcohols, including ethanol, amylose precipitates forming complexes known as Vh-amylose crystals [40]. However, in the same study where SD HASCA was designed, it was suggested that the presence of a Vh form of HASCA was unnecessary to obtain sustained drug-release and that its concentration did not influence the drug-release process, as long as it remained a minor component in a mainly amorphous matrix [6]. Further studies on the complex interactions between ethanol and the solubility of the SR excipients are required to explain the slower release rate. Although the solubility of tramadol hydrochloride is pH independent, distinct solubility of tramadol hydrochloride in a pH gradient versus that in the ethanolic medium, if any, will also influence its release from the formulations.

Tramadol hydrochloride is available from a number of companies as once-daily SR formulations in strengths up to 300 mg. The higher dose in SD HASCA formulations is 200 mg. There is a potential of dose dumping with these formulation if they are tampered with: that is, cut, broke, crushed, chew or simultaneously administered with alcohol. Dose dumping could lead to harmful adverse effects like nausea, dizziness, vomiting, headache, and abdominal pain [41]. Besides, tramadol adverse effects are accentuated in the presence of alcohol.

The influence of ethanol on the* in vitro* release of some opioid drugs, including tramadol hydrochloride, from a number of SR formulations employing diverse release technologies has been investigated following the withdrawn of a once-daily formulation of an opioid drug from the US market. Studies had shown that simultaneous ingestion of ethanol modified its release characteristics and induced dose-dumping, which urged the FDA to consider the issue of potential and harmful changes of the release characteristics of new and marked extended-release formulations caused by ethanol ingestion [25].

**Figure 6:**
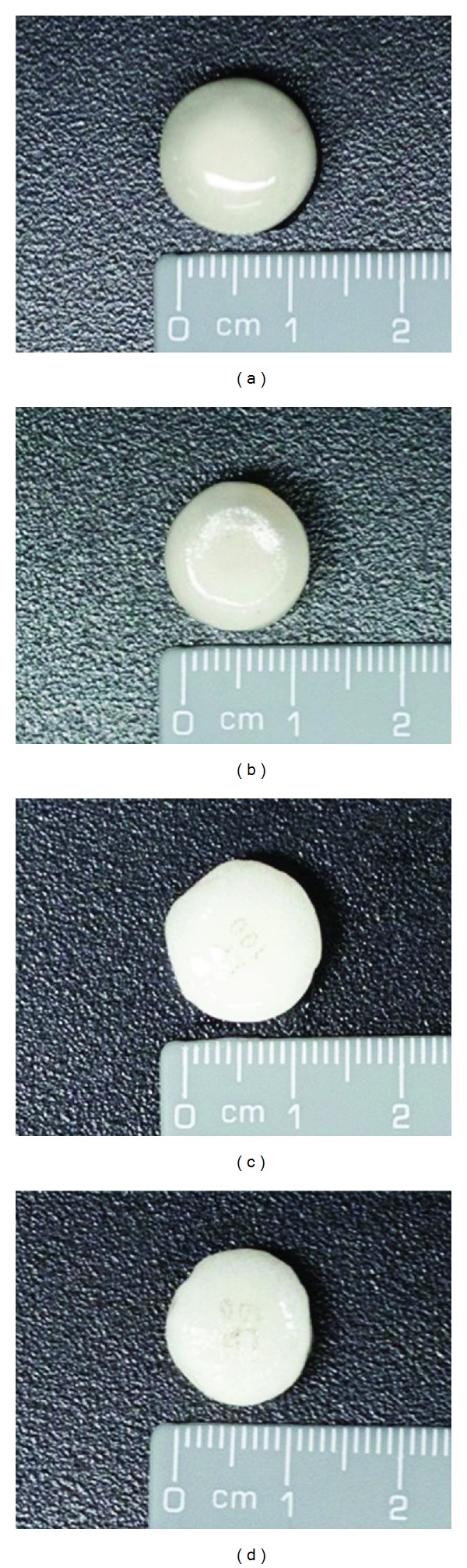
700-mg tablets with 100 mg of tramadol hydrochloride in (a) pH gradient and (b) 40% ethanol hydroalcoholic medium (b); Tridural 100 mg in (c) pH gradient and (d) 40% ethanol hydroalcoholic medium.

The dissolution studies showed that SD HASCA tablets formed a hard gel, which maintained SR properties and was resistant to exposure to a simulation of the pH variations through the gastrointestinal tract as well as to ethanolic conditions. Both SD HASCA and commercial formulations formed a denser gel in ethanolic medium, although the diameter and appearance of the formulations after 7 hours release did not change much (Figure 6). Moreover, the moderate cracks that appeared on the tablet surface during the dissolution tests in a pH gradient did not affect drug-release, which suggests that the risk of undesired burst release* in vivo* might be low.

In fact, an* in vivo* study where SD HASCA tablets containing acetaminophen and sodium chloride were administered to fasted, healthy human volunteers suggested that the formed gel is strong enough to maintain its integrity while traversing the stomach, resisting the vigorous peristaltic contractions which, in the fasted state, clear the stomach of any residual material (commonly known as housekeeper waves) [16]. However, this* in vivo* study was carried out on a very small number of volunteers, and thus its results have to be considered carefully. Moreover, the very high crushing strengths observed for these tablets are likely to prevent possible accidental breaking and crushing. These findings support the value of SD HASCA as a robust excipient for oral sustained drug-release, which may reduce the likelihood of severe adverse effects that can occur in the case of an accidental dose dumping due to misuse (breaking, crushing, bisection of the tablets, or simultaneous alcohol ingestion) of the medication.

## 4. Conclusion

SD HASCA is an interesting excipient for sustained drug-release in solid oral dosage forms. It is biodegradable, inexpensive, and allows facile manufacturing of tablets by direct compression. Tablets made from SD HASCA show independence of drug-release from CF, high crushing strengths, and are amenable to twice-daily and once-daily formulations. The mechanical strength of the SD HASCA gel formed when the tablets are exposed to either a pH gradient or to an ethanolic medium suggests that these tablets are likely to withstand the mechanical stresses that occur after oral administration as well as coadministration with alcohol, preventing undesired disintegration of the tablets in the gastrointestinal tract with consequent dose dumping. Moreover, the properties exhibited by SD HASCA tablets suggest this polymer is a promising and robust excipient for oral sustained drug-release, which may possibly minimize the likelihood of dose dumping and consequent adverse effects, if inadvertent or deliberate misuse of the medication, such as simultaneous ingestion of alcohol, occurs.

## Figures and Tables

**Table 1:** SD HASCA tablets formulation, press used, target CF, and thickness and selected twice-daily and once-daily tablets.

TW (mg)	% of drug (w/w)	Tablet press	Target CF (tons/cm^2^), HP	Target thickness (mm), SSP	Selected twice-daily and once-daily tablets
400	12.5	HP	2.5	—	—
	25	HP	2.5	—	—
	37.5	HP	2.5	—	—
	50	HP	2.5	—	—
	60	HP	2.5	—	—

450	22.22	HP	2.5	—	Twice-daily (not selected)

500	20	HP	2.5	—	—
	30	HP	2.5	—	—
	40	HP	2.5	—	Twice-daily (not selected)
	50	HP	2.5	—	—
	60	HP	2.5	—	—

700	14.3	HP	1	—	—
	14.3	HP	1.5	—	—
	14.3	HP	2	—	—
	14.3	HP	2.5	—	Once-daily (not selected)

800	12.5	HP	2.5	—	—
	25	HP	2.5	—	Once-daily (not selected)
	37.5	HP	2.5	—	—
	50	HP	2.5	—	—
	60	HP	2.5	—	—

**450**	**22.22 (100 mg)**	**SSP**	—	**4.63**	**Twice-daily (selected)**
**500**	**40 (200 mg)**	**SSP**	—	**5.15**	**Twice-daily (selected)**
**700**	**14.3 (100 mg)**	**SSP**	—	**6.98**	**Once-daily (selected)**
**800**	**25 (200 mg)**	**SSP**	—	**7.43**	**Once-daily (selected)**

**Table 2:** Time to release 50% (T50%) and 90% (T90%) of drug from SD HASCA tablets with total weights between 400*** ***mg and 800*** ***mg and % of drug (w/w) between 12.5% and 60%.

TW (mg)	[T50%, T90%] (h)	% of drug (w/w)									
		12.5	14.3	20	22.22	25	30	37.5	40	50	60
400	T50%	1.8	—	—	—	1.5	—	1.4	—	1.3	1.1
	T90%	6.5	—	—	—	5.3	—	4.5	—	4.6	3.5
450	T50%	—	—	—	1.9	—	—	—	—	—	—
	T90%	—	—	—	6.4	—	—	—	—	—	—
500	T50%	—	—	2.55	—	—	2.05	—	1.9	1.75	1.4
	T90%	—	—	8.75	—	—	7.6	—	6.7	5.9	4.65
700	T50%	—	4	—	—	—	—	—	—	—	—
	T90%	—	14.4	—	—	—	—	—	—	—	—
800	T50%	4.9	—	—	—	4.1	—	3.3	—	2.7	2.5
	T90%	18.4	—	—	—	16.4	—	11.5	—	10.25	9.3

**Table:** (a) Frequency of administration: twice-daily

Formulation				
Commercial			SD HASCA (SSP)	
Topalgic LP 100 mg	Topalgic LP 200 mg	Contramal LP 200 mg	100 mg of drug (SSP)	200 mg of drug (SSP)
[2.15, 7.60]	[2.00, 6.90]	[2.10, 6.85]	[3.00, 10.70]	[3.00, 10.25]

**Table:** (b) Frequency of administration: Once-daily

Formulation					
Commercial				SD HASCA (SSP)	
Tridural 100 mg	Tridural 200 mg	Ralivia 200 mg	Zytram XL 200 mg	100 mg of drug (SSP)	200 mg of drug (SSP)
[6.10, 15.70]	[6.00, 19.50]	[6.40, 11.60]	[7.50, >24]	[5.10, 20.40]	[5.20, 19.10]

**Table 4:** Time to release 50% and 90% of tramadol hydrochloride [T50%, T90%] for SD HASCA tablets compressed between 1 and 2.5 tons/cm^2^ and similarity factors (*f*_2_) between tablets weighting 700-mg tablets with 100*** ***mg of tramadol hydrochloride, compressed with a 30-tons manual HP at different CFs.

CF (tons/cm^2^)	[T50%, T90%] (hours)	*f*_2_ versus 2.5 tons/cm^2^
1	[3.90, 14.25]	90
1.5	[4.10, 14.50]	89
2	[4.25, 15.40]	84
2.5	[4.00, 14.40]	

**Table 5:** Time to release 50% and 90% (hours) of tramadol hydrochloride [T50%, T90%] from twice-daily and once-daily SD HASCA tablets with 100 mg and 200 mg of drug, compressed using a 30-tons manual hydraulic press at 2.5 tons/cm^2^ (HP) or a single-stroke press (SSP).

Frequency of administration	Formulation			
	100 mg of drug (HP)	100 mg of drug (SSP)	200 mg of drug (HP)	200 mg of drug (SSP)
Twice-daily	[1.90, 6.40]	[3.00, 10.70]	[1.90, 6.70]	[3.00, 10.25]
Once-daily	[4.00, 14.40]	[5.20, 20.40]	[4.10, 16.40]	[5.20, 19.10]
